# Formulation of Nanomicelles Loaded with Cannabidiol as a Platform for Neuroprotective Therapy

**DOI:** 10.3390/pharmaceutics14122625

**Published:** 2022-11-28

**Authors:** Yordan Yordanov, Denitsa Stefanova, Ivanka Spassova, Daniela Kovacheva, Virginia Tzankova, Spiro Konstantinov, Krassimira Yoncheva

**Affiliations:** 1 Department of Pharmacology, Pharmacotherapy and Toxicology, Faculty of Pharmacy, Medical University of Sofia, 1000 Sofia, Bulgaria; 2 Institute of General and Inorganic Chemistry, Bulgarian Academy of Sciences, 1113 Sofia, Bulgaria; 3 Department of Pharmaceutical Technology and Biopharmaceutics, Faculty of Pharmacy, Medical University of Sofia, 1000 Sofia, Bulgaria

**Keywords:** nanomicelles, cannabidiol, neuroblastoma cells, neuroprotection

## Abstract

The present study is focused on the development of cannabidiol-loaded polymeric nanomicelles as a drug delivery system with neuroprotective effects. Cannabidiol was loaded in Pluronic micelles (Pluronic P123 or its combination with Pluronic F127) possessing an average diameter smaller than 50 nm and high encapsulation efficiency for the hydrophobic drug (80% and 84%, respectively). The successful encapsulation and transformation of cannabidiol in amorphous phase were observed by IR spectroscopy and X-ray diffraction, respectively. Studies with neuroblastoma cells (SH-SY5Y and Neuro-2a) showed that the pure cannabidiol caused a dose-dependent reduction of cell viability, whereas its loading into the micelles decreased cytotoxicity. Further, neuroprotective effects of pure and micellar cannabidiol were examined in a model of H_2_O_2_-induced oxidative stress in both neuroblastoma cells. The pre-treatment of cell lines with cannabidiol loaded into the mixed Pluronic P123/F127 micelles exerted significantly stronger protection against the oxidative stress compared to pure cannabidiol and cannabidiol in single Pluronic P123 micelles. Interestingly, the empty mixed P123/F127 micelles demonstrated protective activity against the oxidative stress. In conclusion, the study revealed the opportunity to formulate a new drug delivery system of cannabidiol, in particular nanosized micellar aqueous dispersion, that could be considered as a perspective platform for cannabidiol application in neurodegenerative diseases.

## 1. Introduction

Neuronal cell death is a hallmark of many diseases of the central nervous system, including neurodegenerative disorders, acute neurotoxicant exposure, and different types of damage to the brain and spinal cord. Oxidative stress and inflammation are key contributors to neuronal loss by either directly causing neuronal cell death or activating a cascade of cellular events, which eventually result in apoptosis [1]. However, attempts at discovering effective therapeutic options for neurodegenerative disorders have so far failed [2]. Cannabidiol (CBD) is a natural substance, known for its pleiotropic antioxidant and anti-inflammatory effects. Furthermore, it has antiepileptic, anxiolytic, and analgesic activity and lacks the psychoactive effects and dependence typical for tetrahydrocannabinol [3]. CBD is the active constituent of the registered Epidyolex, used in the management of seizures, related to Dravet syndrome, Lennox–Gastaut syndrome, or tuberous sclerosis [4]. Although CBD’s mechanism of action remains unclear, it is believed that its effects are induced via receptor action. CBD has affinity to cannabinoid receptors, transient receptor potential (TRP) channels, and nuclear receptors, such as peroxisome proliferator-activated receptors (PPARγ) [5]. PPARγ promotes the cellular antioxidant defense and there may be a link between TRP, calcium homeostasis, and neuronal loss [6]. CBD could also possibly promote adult neurogenesis by CB_1_R and CB_2_R and brain-derived neurotrophic factor (BDNF) signaling modulation [7,8]. The predominant mechanisms by which CBD is believed to exert its antioxidant effects are via stimulation of the cellular antioxidant systems through activating nuclear factor erythroid 2-related factor 2 (Nrf-2), by inhibiting the nuclear factor kappa-light-chain-enhancer of activated B cells (NFκB) pathway [9], as well as via its direct antioxidant activity [5,10]. 

CBD is classified as a Class II drug according to the Biopharmaceutics Classification System, meaning that it is a poorly soluble and highly permeable drug substance. Despite its plethora of pharmacological effects, including neuroprotection, the application of CBD is limited by its lack of aqueous solubility (<5 μg/mL), problematic first-pass effect, and poor bioavailability of approximately 6% in humans [11,12]. To date, the main formulations for oral administration of CBD are oil and alcoholic solutions, soft capsules, and oromucosal spray. However, the limitations discussed above emphasize the importance of developing new drug delivery systems which are able to improve the solubility of CBD and preserve its biological activity. For example, a comparison between CBD oil solution and nano-emulsion demonstrated that the formulation of CBD in nano-emulsion improved its bioavailability after oral administration in rats (AUC_0–∞_/dose) by approximately 65% [13]. Interestingly, another study revealed that lipid nanocapsules loaded and decorated with CBD actively target glioma cells [14]. A very attractive strategy for efficient CBD delivery is to develop new drug systems that would be able to replace the oil vehicles. In this view, the self-association of biocompatible amphiphilic copolymers in aqueous medium can be applied to the formulation of CBD in micellar dispersions. Sosnik et al. [15] have loaded approximately 20% cannabidiol in mixed micelles based on chitosan/poly(vinyl alcohol) and poly(methyl methacrylate) and observed improvement of its trans-corneal delivery. A recent study incorporated CBD into micelles of polysaccharide fucoidan and deoxycholic acid [16]. The authors registered a stronger anti-inflammatory effect of the micellar CBD compared to the pure drug after local or systemic in vivo administration. Among various amphiphilic copolymers, the triblock copolymers of poly(ethylene oxide) and poly(propylene oxide) (PEO–PPO–PEO) are well-known micellar carriers possessing a high capacity for loading of hydrophobic molecules [17,18]. In addition, the size of PEO–PPO–PEO (available as Pluronics, Poloxamers) micelles is typically smaller than 50 nm. Thus, the small size would increase the surface area, and as a result, the solubility rate of the included drug in the aqueous physiological fluids. Rao et al. [19] developed CBD-loaded nanomicelles based on such a copolymer (Poloxamer 407). The incorporation of CBD in these micelles exerted higher bioavailability and a stronger anti-inflammatory effect in mice compared to administration of pure CBD. 

The aim of the present study was to evaluate the potential of nanosized micellar formulations as a platform for neuronal delivery of CBD. The drug was loaded in Pluronic micelles, and the physicochemical characteristics and stability of the developed micelles were examined. The influence of pure and micellar CBD on the characteristics and viability of two neuroblastoma cell lines (SH-SY5Y and Neuro-2a) was studied by the MTT assay and flow-cytometry. Further, the antioxidant effects of pure and micellar CBD were investigated in a model of oxidative stress, aiming to consider the potential of the micellar system in neuronal therapy.

## 2. Materials and Methods

### 2.1. Materials

Cannabidiol (CBD) was donated by PBG Global Ltd. (Sofia, Bulgaria). The copolymers Pluronic P123 (PEO_20_PPO_70_PEO_20_) and Pluronic F127 (PEO_101_PPO_56_PEO_101_) were provided by Sigma-Aldrich (Steinheim, Germany). The cell culture medium RPMI, heat-inactivated fetal bovine serum (FBS), L-glutamine, 3-(4,5-dimethylthiazol-2-yl)-2,5-diphenyltetrazolium bromide (MTT), hydrogen peroxide, and dimethyl sulfoxide (DMSO) were purchased from Sigma-Aldrich (Merck KGaA, Darmstadt, Germany). The human neuroblastoma cell line SH-SY5Y and the mouse neuroblastoma cell line Neuro-2a were obtained from the European Collection of Cell Cultures (ECACC, Salisbury, UK). The cells were grown in the complete RPMI 1640 medium, which contained L-glutamine (2 mM) and 10% heat-inactivated fetal bovine serum. Both cell lines were maintained in 75 cm^2^ flasks, in a humidified, 5% CO_2_, 37 °C incubator (Esco, CCl-170B-8, Singapore).

### 2.2. Loading of CBD in Pluronic Micelles

CBD-loaded micelles were prepared by the film hydration method using methanol or ethanol as solvents. The copolymers, Pluronic P123 or the mixture of Pluronic P123 and Pluronic F127 (1:1, wt/wt), were dissolved in the selected solvent (10 mg/mL) under stirring (700 rpm). After that, CBD was added to the respective polymeric solution, resulting in a concentration of 1 mg of CBD per mL. The organic solvent was evaporated overnight under magnetic stirring. The resulted film was dispersed in purified water, affording aqueous micellar dispersion. The dispersions were filtered (0.22 µm) and the concentration of unloaded CBD was determined in the ethanolic fractions (50%) used for filter rinsing. The amount of CBD in the fractions was measured by UV-visible spectrophotometry (Thermo Fisher Scientific) at 275 nm [20]. The concentration of CBD was calculated according to a standard curve prepared in 50% ethanol (0.032–0.32 mg/mL, r > 0.9959). The encapsulation efficiency (EE) was calculated using the equation:

EE = (initial amount of drug − non-loaded drug)/initial amount of drug

### 2.3. Physicochemical Characterization of the Micelles

The size, polydispersity index, and zeta-potential of empty and CBD-loaded micelles were evaluated by photon correlation spectroscopy and electrophoretic laser doppler velocimetry (Zetamaster Analyzer, Malvern Instruments, UK). The samples of micellar dispersions were diluted with distilled water 5 times and their characteristics were measured at 25 °C with a scattering angle of 90°.

The stability of the micellar dispersions was evaluated by determination of micellar size and drug loading immediately after preparation and after 30 days of storage. CBD-loaded micelles were stored in glass vials that were incubated at 25 °C. 

IR spectra were recorded with a Nicolet Avatar 360 FTIR spectrometer with accumulation of 64 scans and spectral resolution of 2 cm^−1^ in the spectral range of 400 to 4000 cm^−1^. The samples were prepared in KBr pellets.

Powder X-ray diffraction (PXRD) patterns were obtained in the range 5–60° 2θ, on a Bruker D8-Advance Diffractometer equipped with a Cu tube (λ = 1.5418 Å) and a LynxEye detector. The evaluation of the diffraction patterns was performed using the DiffracPlus EVA software package in combination with the ICDD-PDF-2 database.

### 2.4. Cell Viability

The SH-SY5Y and Neuro-2a cells were plated for 24 h at 37 °C on 96-well plates at a density of 2 × 10^4^ cells per well, to confluency. After 24 h, the cells were treated with pure cannabidiol (CBD) at concentrations between 0.4 and 100 µM, and the corresponding concentrations of CBD loaded in P123 (CBD-P123) and mixed (CBD-P123/F127) micelles. The cells were incubated with the test solutions for 24, 48, and 72 h, and the MTT solution (10 mg/mL in PBS) was then added to each well and the mixture was incubated at 37 °C for 3 h. Thereafter, the MTT solution was carefully aspirated, and the obtained formazan crystals were dissolved by the addition of 100 μL of DMSO. The absorbance was measured in a multiplate reader, Synergy 2 (BioTek Instruments, Inc., Highland Park, Winooski, VT, USA), at 570 nm (690 nm for background absorbance).

### 2.5. Flow-Cytometric Analysis

Treated and control cells were trypsinized, centrifuged, washed, and resuspended in PBS and stained with propidium iodide (50 µg/mL), according to a previously described protocol [21]. After 1 h of incubation at 4 °C, at a place protected from light, cell suspensions were analyzed on a FACS Canto II flow-cytometer at the excitation wavelength of 488 nm. At least 20,000 events were detected for each sample. Dot plots of the obtained data were gated and the limits between cell cycle phases and apoptotic cells were uniformly specified on all resulting subpopulation histograms. 

### 2.6. In Vitro Model of H_2_O_2_-Induced Oxidative Stress

The cells were seeded in 96-well plates at appropriate density (3.5 × 10^4^ cells for SH-SY5Y and 3.0 × 10^4^ cells for Neuro-2a) and allowed to attach at the well’s bottom for 24 h. Then, they were pretreated with solutions of pure and micellar CBD (final concentrations: 0.22, 0.39, and 0.78 µM) and empty micelles (concentrations of P123 micelles: 4.75, 8.47, 19, and 38 µg/mL, and mixed P123/F127 micelles: 4.5, 8, 18, and 36 µg/mL) in RPMI for 90 min before H_2_O_2_ exposure. Afterwards, the cells were washed with phosphate-buffered saline (PBS) to remove the extracellular amount of the test solutions. Subsequently, SH-SY5Y cells were subjected to 1 mmol/L of H_2_O_2_ in PBS for 10 min, and Neuro-2a cells were treated with 3 mmol/L of H_2_O_2_ in PBS for 10 min. Then, the contents of the wells were exchanged with culture medium. After 24 h, the amount of the attached, viable cells was evaluated by the MTT assay. Negative controls (not treated with hydrogen peroxide) were considered a measure for 100% protection, and positive controls (hydrogen peroxide-treated) for 0% protection.

### 2.7. Statistical Analysis

Statistical analysis was performed using GraphPad Prism 6 Software. Control and treatment group data were compared using one-way ANOVA and Dunnett’s multiple comparisons post-test. A significance level of 0.05 was chosen for all comparisons.

## 3. Results and Discussion

**Figure 1:**
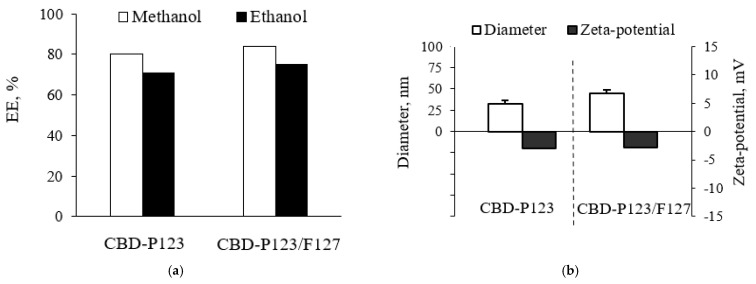
Encapsulation efficiency depending on the applied solvent (**a**) and physicochemical characteristics (**b**) of cannabidiol-loaded Pluronic P123 and mixed P123/F127 micelles.

This study is focused on the formulation of CBD into micellar drug delivery systems, aiming to obtain stable aqueous nano-dispersion of CBD. Such formulation would be advantageous taking in consideration the limited application of CBD because of its poor water solubility. For example, CBD inhibits SARS-CoV-2 replication, but due to the low solubility, the drug was intraperitoneally administered in mice as an injection solution containing ethanol, Cremophor EL, and phosphate buffer [22]. Many studies have reported successful loading of hydrophobic drugs in Pluronic micelles and consecutive improvement of drug efficacy through micellar systems [23,24,25]. In the present study, two types of Pluronic micelles were selected as water-dispersible nanocarriers for CBD—single Pluronic P123 micelles (P123) and mixed micelles based on Pluronic P123 and Pluronic F127 (P123/F127). Both types of micelles were loaded with CBD, applying a film hydration method, which showed high efficiency for loading of hydrophobic drugs [26,27,28]. For example, Liu et al. [26] achieved a 92% encapsulation efficiency of docetaxel in Pluronic P123 micelles applying the film hydration method. In our study, the use of methanol as a solvent resulted in a high CBD encapsulation efficiency, in particular 80% in P123 micelles and 84% in mixed P123/F127 micelles. Methanol was considered as a more appropriate solvent because with ethanol, the efficiency was lower, 71% and 75%, respectively (Figure 1a). In our opinion, a possible reason for the successful encapsulation was probably the hydrophobic interactions between the drug, which is highly hydrophobic (logP 6.3), and the PPO-chains in the micellar core. The resulted CBD-loaded Pluronic P123 micelles were characterized with a mean diameter of 32 nm, whereas the mixed micelles possessed a slightly larger diameter of 45 nm (Figure 1b). The polydispersity showed the same trend, in particular a slightly lower index of Pluronic P123 micelles compared to the mixed P123/F127 micelles (0.259 and 0.291, respectively). Thus, the properties of the resulted micelles were considered appropriate for a drug delivery system deliberating that the small size enables nanoparticle transport through the cellular membranes [29,30,31]. Both the small size of the micelles and the surface-active properties of the carriers are a prerequisite for efficient intracellular uptake [32]. The values of zeta-potential were slightly negative, which confirmed previous observations for Pluronic-based micelles [33,34]. 

**Figure 2:**
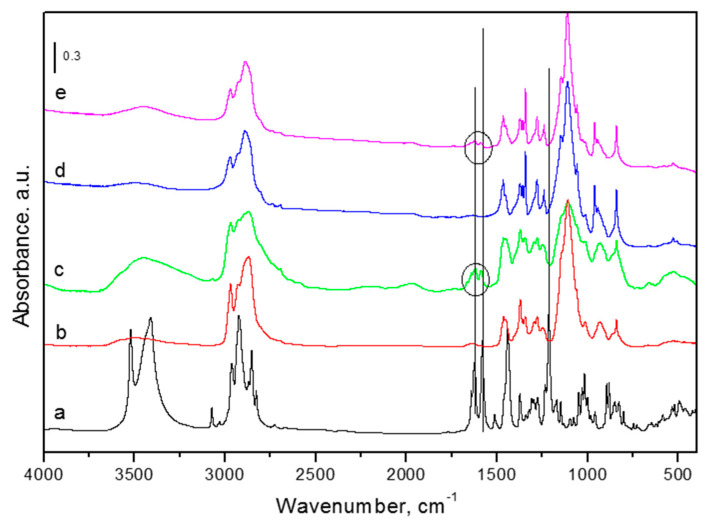
IR spectra of pure cannabidiol (a), empty Pluronic P123 micelles (b), CBD-loaded Pluronic P123 micelles (c), empty (d), and CBD-loaded mixed Pluronic P123/F127 micelles (e).

The successful encapsulation of CBD into both types of micelles was considered taking in account the appearance of characteristic peaks of CBD in IR spectra of loaded formulations (Figure 2). The most intense peaks in the spectrum of pure CBD (Figure 2a) were at 3412 and 3522 cm^−1^, corresponding to the O–H stretching, the band at ~3070 cm^−1^ was assigned to C–H stretching (phenyl), ~2980–2820 cm^−1^ was indicative of methyl and methylene groups, 1625 and 1580 cm^−1^ were attributed to C=C stretching (phenyl ring) and =C–H, while C–O stretching vibrations were recorded at ~1214 cm^−1^ [35,36]. In the FTIR spectra of the drug-loaded micelles (CBD-P123 and CBD-P123/F127), two characteristic bands of CBD at 1580 and 1625 cm^−1^, confirming the presence of C=C (phenyl ring) and =C–H aliphatic groups in the formulations, were preserved. On the other hand, the disappearance of the most intense peak at 1214 cm^−1^ in both drug-loaded micelles could be an indication for an interaction between CBD and carriers [35]. Bands at 938 and 839 cm^−1^ in all spectra were related to vibrational modes of CH_2_ and C-O-C groups [37]. The band at ~3070 cm^−1^ was visible only in the spectrum of CBD-P123 but not in the spectrum of CBD-P123/F127, which suggests a stronger interaction of CBD with the P123/F127 micelles. 

**Figure 3:**
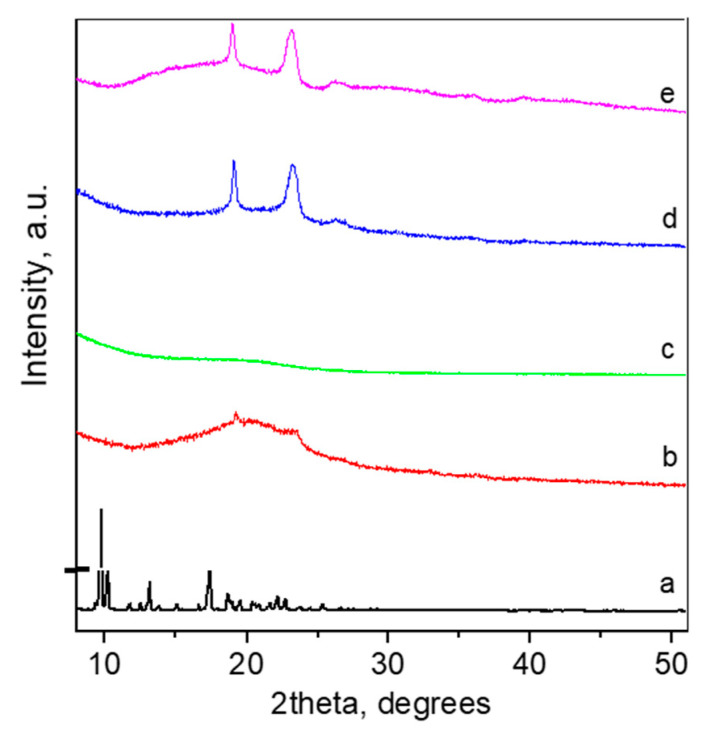
XRD pattern of pure cannabidiol (a), empty Pluronic P123 micelles (b), CBD-loaded Pluronic P123 micelles (c), empty (d), and CBD-loaded mixed Pluronic P123/F127 micelles (e).

XRD analyses showed that CBD crystallizes in monoclinic SG P2_1_ and the profile fit results with the refined unit cell parameters, as follows: a = 10.627(2) Å, b = 10.629(1) Å, c = 17.298(2) Å, and β = 95.39(2)°. These values were close to those reported in the literature [38]. The crystallite size was >200 nm, indicating high crystallinity of the initial compound. The XRD pattern of CBD loaded in P123 micelles led to changes of both the loaded drug and the micelles (Figure 3b,c). Both structures were completely destroyed, and an amorphous pattern was seen as a result. On the other hand, the loading of CBD into the mixed P123/F127 micelles revealed that the crystallinity of CBD totally deteriorated and a hump typical for amorphous phase appeared. XRD did not reveal changes in the pattern of empty mixed micelles, which means that their structures remained unchanged (Figure 3d,e). 

**Figure 4:**
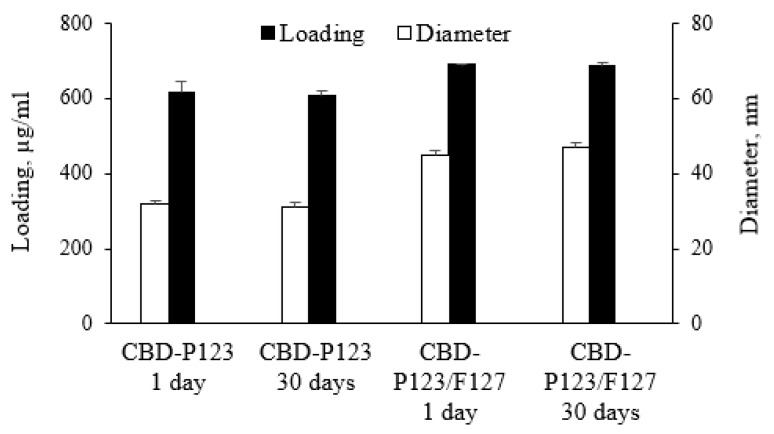
Stability of micellar formulations regarding the mean diameter of micelles and drug loading.

The stability of the resulted micellar dispersions was examined by determination of micellar size and CBD loading (Figure 4). The results showed that both parameters did not change over 30 days of storage, which indicated that there was no drug leakage or aggregation of micelles. In addition, the values of the polydispersity index and zeta-potential did not change, which indicated the high colloidal stability of the micellar dispersion. In our opinion, the slightly negative zeta-potential generates steric repulsion, which overcomes aggregation of the micelles. A similar high stability of micelles formulated with Pluronic F127 was reported in recent studies [39,40]. 

**Figure 5:**
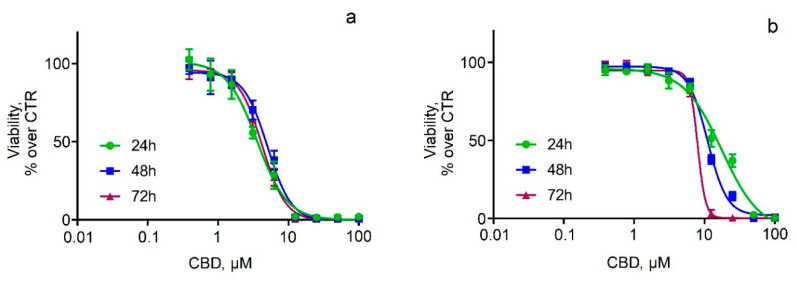
Cytotoxic effects after treatment of SH-SY5Y cells (**a**) and Neuro-2a cells (**b**) with pure CBD. Values are presented as the percentage of untreated controls and expressed as mean ± SD (*n* = 6).

**Figure 6:**
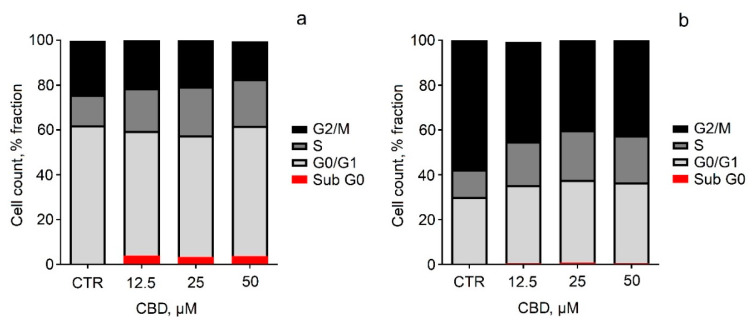
Distribution by cell cycle phase of SH-SY5Y cells (**a**) and Neuro-2a cells (**b**) after 24 h of treatment with different concentrations of pure CBD. Gap 1/quiescent phase (G0/1), synthesis phase (S), gap 2/mitosis phase (G2/M), and apoptotic cells (sub-G0). Values are presented as the percentage of the cell population in studied samples.

**Figure 7:**
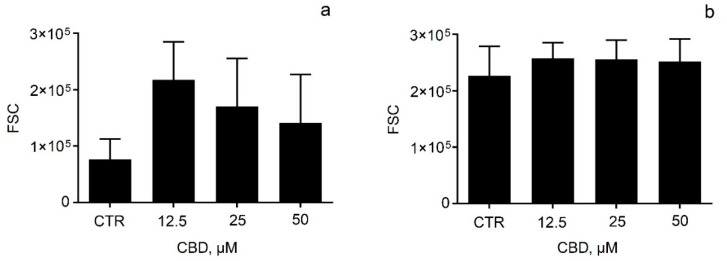
Comparative evaluation of the diameter of SH-SY5Y (**a**) and Neuro-2a (**b**) cells by forward light scatter (FSC) flow-cytometric evaluation after 24 h of treatment with pure CBD. Data are presented as mean ± SD.

The next task of our study was to examine the viability of two neuroblastoma cell lines (SH-SY5Y and Neuro-2a) after treatment with pure and micellar CBD. First, cell viability studies showed that pure CBD caused dose-dependent cytotoxicity, to which SH-SY5Y cells were more susceptible (Figure 5a). These observations were quantified with IC_50_ values, which were about 3 to 5 µM for SH-SY5Y cells and 8 to 17 µM for Neuro-2a cells, indicating that the viability of Neuro-2a cells was time-dependent (Figure 5b). To understand the effect of CBD on cell cycle phase distribution, flow-cytometric analysis of unfragmented DNA content in single cells after propidium iodide staining was performed. The cells were treated with pure CBD in concentrations at which it caused pronounced effects on cell viability (12.5, 25, and 50 μM). The results were expressed as the percentages of cells in each cell cycle phase (Figure 6). Some differences were observed in cell cycle distribution between both neuroblastoma cell lines. In the SH-SY5Y cell line, G0/G1 cells were the major fraction, while in the Neuro-2a line, cells in G2/mitosis predominated (Figure 6). Treatment of SH-SY5Y cells with CBD (12.5–50 μM) increased the number of apoptotic cells and cells in the synthesis phase at the expense of the fraction of G2/M cells (Figure 6a). Neuro-2a cells also underwent changes in cell cycle phase upon CBD treatment, but the increase of apoptotic cells was negligible, and the decrease of G2/M cells was more obvious compared to the treated SH-SY5Y cells (Figure 6b). In in vitro conditions, the initial normotonic cell shrinkage was followed by necrosis and an increase of diameter due to the lack of phagocytes. We evaluated the changes in cell diameter by FSC flow-cytometric evaluation after CBD treatment (Figure 7). The observed increase of sub-G0 apoptotic cells was accompanied by an increase of the cell diameter (Figure 7a). In Neuro-2a cells, where apoptosis was not significant, the increase of the cell diameter was also negligible (Figure 7b).

**Figure 8:**
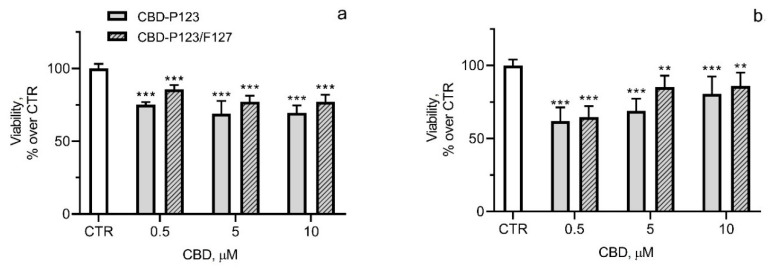
Effects on cell viability after 72 h of treatment of SH-SY5Y cells (**a**) and Neuro-2a cells (**b**) with CBD loaded in P123 or mixed P123/F127 micelles. Values are presented as the percentage of untreated controls and expressed as mean ± SD (*n* = 6). ** *p* < 0.01, *** *p* < 0.001 vs. control.

The effects of CBD loaded in both types of micelles on the cell viability of SH-SY5Y and Neuro-2a cells are presented in Figure 8. In general, the treatment with CBD loaded into P123 and mixed P123/F127 micelles increased cell viability compared to the non-loaded CBD (Figure 5a and Figure 8a). This effect was more pronounced with the mixed P123/F127 micelles. The treatment of SH-SY5Y cells with CBD-P123/F127 micelles (corresponding to 5 and 10 μM CBD) maintained about 77% cell viability, whereas with pure CBD it was about 37% and 8%, respectively (Figure 5a and Figure 8a). In Neuro-2a cells, the viability of the cells after treatment with CBD-P123/F127 micelles approximated 86%, whereas the pure CBD reduced cell viability to almost 14% (Figure 5b and Figure 8b). 

**Figure 9:**
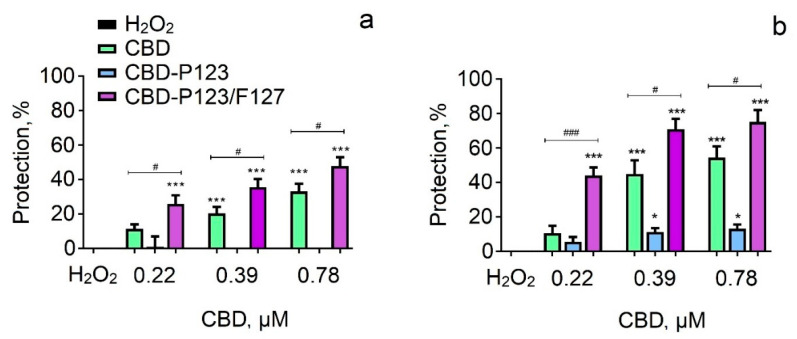
Protective effects of pure CBD and CBD loaded in P123 and mixed P123/F127 micelles in a model of H_2_O_2_-induced oxidative damage in SH-SY5Y (**a**) and Neuro-2a (**b**) cells. Data are presented as means from three independent experiments ± SD (*n* = 8). * *p* < 0.05, *** *p* < 0.001, vs. H_2_O_2_ group (one-way analysis of variance with Dunnett’s post hoc test). Comparisons between corresponding concentrations of pure and micellar CBD were performed with multiple T-tests, corrected by the Holm–Sidak method. # *p* < 0.05, ### *p* < 0.001, vs. CBD.

The antioxidant effects of pure CBD and CBD loaded in P123 and mixed P123/F127 micelles were evaluated in a model of H_2_O_2_-induced oxidative stress in both neuroblastoma cell lines. The incubation with H_2_O_2_ led to a significant cell viability loss in SH-SY5Y cells, thus confirming its toxicity (Figure 9a). The pre-incubation with the empty micelles showed no protection on neuronal cells (results not shown), whereas the pre-incubation of SH-SY5Y cells with pure CBD induced statistically significant protection at a concentration of 0.39 and 0.78 μM, resulting in preservation of cell viability by 20% and 33% vs. H_2_O_2_. The pretreatment with CBD loaded in P123 micelles did not show statistically significant protection at all tested concentrations. In contrast, the pretreatment of cells with CBD loaded into the mixed micelles (CBD-P123/F127) showed evidently cytoprotective effects at all tested concentrations. Preservation of cell viability was observed with 26%, 36%, and 48% vs. H_2_O_2_, respectively. Furthermore, it is important to note that the protection with the micellar CBD was more pronounced than the protection with pure CBD. Preservation of cell viability was detected even at the lowest concentration of CBD into the mixed P123/F127 micelles (0.22 μM), in which pure CBD was not protective at all (Figure 9a). 

In the next series of experiments, pretreatment was performed with pure and micellar CBD on the Neuro-2a cell line. Comparing to SH-SY5Y cells, it could be noted that Neuro-2a cells were more sensitive, since the antioxidant effects of pure and micellar CBD were stronger than those on SH-SY5Y cells (Figure 9b). The results showed that incubation of Neuro-2a cells with pure CBD provoked statistically significant protective effects at concentrations of 0.39 and 0.78 µM, maintaining cell viability with 45% and 54% vs. H_2_O_2_. The pretreatment with CBD loaded into P123 micelles showed significantly weaker protection than that of the pure CBD. In contrast, the pretreatment of cells with CBD loaded into P123/F127 micelles showed significant cytoprotective effects at all tested concentrations. The protection with this drug system was more pronounced than the protection achieved with the pure CBD. The lowest concentration of pure CBD did not show any protection on Neuro-2a cells, whereas CBD loaded in P123/F127 micelles showed protection of 44% (Figure 9b). Interestingly, the pretreatment of Neuro-2a cells with the empty mixed P123/F127 micelles at concentrations of 8, 18, and 36 µg/mL demonstrated protection against the oxidative stress (results not shown), which additionally contributed to the stronger protection with this formulation of cannabidiol. 

## 4. Conclusions

The present study outlined a new strategy for the inclusion of cannabidiol in a micellar drug delivery system based on Pluronic copolymers. The mixed P123/F127 micelles provided high encapsulation efficiency for the hydrophobic drug (84%). Cannabidiol loading in these micelles resulted in lower drug cytotoxicity on neuronal cells and stronger neuroprotective effects against induced oxidative stress. More importantly, at a low concentration (0.22 µM), pure cannabidiol had no protective effect against cellular oxidative stress, whereas the drug loaded in P123/F127 micelles increased the viability of SH-SY5Y and Neuro-2a neuronal cells by 26% and 44%, respectively. Thus, the proposed micellar system could be a perspective platform to develop new cannabidiol dosage forms for therapeutic interventions in neurodegenerative diseases.

## Data Availability

Not applicable.

## References

[1] Fricker M., Tolkovsky A.M., Borutaite V., Coleman M., Brown G.C. (2018). Neuronal cell death. Physiol. Rev..

[2] Maher P. (2019). The potential of flavonoids for the treatment of neurodegenerative diseases. Int. J. Mol. Sci..

[3] Brunetti P., Lo Faro A.F., Pirani F., Berretta P., Pacifici R., Pichini S., Busardo F.P. (2020). Pharmacology and legal status of cannabidiol. Ann. Ist. Super Sanita.

[4] Abu-Sawwa R., Scutt B., Park Y. (2020). Emerging use of epidiolex (cannabidiol) in epilepsy. J. Pediatr. Pharmacol. Ther..

[5] Atalay S., Karpowicz I., Skrzydlewska E. (2020). Antioxidative and anti-inflammatory properties of cannabidiol. Antioxidants.

[6] Hong C., Jeong B., Park H.J., Chung J.Y., Lee J.E., Kim J., Shin Y.C., So I. (2020). TRP channels as emerging therapeutic targets for neurodegenerative diseases. Front. Physiol..

[7] Ferreira F.F., Ribeiro F.F., Rodrigues R.S., Sebastião A.M., Xapelli S. (2018). Brain-derived neurotrophic factor (BDNF) role in cannabinoid-mediated neurogenesis. Front. Cell. Neurosci..

[8] Sales A.J., Fogaça M.V., Sartim A.G., Pereira V.S., Wegener G., Guimarães F.S., Joca S.R.L. (2019). Cannabidiol induces rapid and sustained antidepressant-like effects through increased BDNF signaling and synaptogenesis in the prefrontal cortex. Mol. Neurobiol..

[9] Jastrząb A., Gęgotek A., Skrzydlewska E. (2019). Cannabidiol regulates the expression of keratinocyte proteins involved in the inflammation process through transcriptional regulation. Cells.

[10] Hamelink C., Hampson A., Wink D.A., Eiden L.E., Eskay R.L. (2005). Comparison of cannabidiol, antioxidants, and diuretics in reversing binge ethanol-induced neurotoxicity. J. Pharmacol. Exp. Ther..

[11] World Health Organization (2017). Expert Committee on Drug Dependence: Cannabidiol (CBD) Pre-Review Report Agenda Item 5.2 and Peer Review. https://www.who.int/medicines/access/controlled-substances/5.2_CBD.pdf.

[12] Perucca E., Bialer M. (2020). Critical aspects affecting cannabidiol oral bioavailability and metabolic elimination, and related clinical implications. CNS Drugs.

[13] Nakano Y., Tajima M., Sugiyama E., Sato V.H., Sato H. (2019). Development of a novel nano-emulsion formulation to improve intestinal absorption of cannabidiol. Med. Cannabis Cannabinoids.

[14] Holgado M.A., Martin-Banderas L., Alvarez-Fuentes J., Fernandez-Arevalo M. (2017). Neuroprotective effect of cannabinoids nanoplatforms in neurodegenerative diseases. J. Drug Deliv. Sci. Technol..

[15] Sosnik A., Shabo R.B., Halamish H.M. (2021). Cannabidiol-loaded mixed polymeric micelles of chitosan/poly(vinyl alcohol) and poly(methyl methacrylate) for trans-corneal delivery. Pharmaceutics.

[16] Liu Y., Qi X., Wang Y., Li M., Yuan Q., Zhao Z. (2022). Inflammation-targeted cannabidiol-loaded nanomicelles for enhanced oral mucositis treatment. Drug Deliv..

[17] Kabanov A.V., Batrakova E.V., Alakhov V.Y. (2002). Pluronic^®^ block copolymers as novel polymer therapeutics for drug and gene delivery. J. Contoll. Rel..

[18] Figueiras A., Domingues C., Jarak I., Santos A.I., Parra A., Pais A., Alvarez-Lorenzo C., Concheiro A., Kabanov A., Cabral H. (2022). New advances in biomedical application of polymeric micelles. Pharmaceutics.

[19] Rao Y., Li R., Liu S., Meng L., Wu Q., Yuan Q., Liang H., Qin M. (2022). Enhanced bioavailability and biosafety of cannabidiol nanomicelles for effective anti-inflammatory therapy. Particuology.

[20] Duarte P. (2016). Determination of the antibiotic properties of cannabidiol. J. Gen. Pract..

[21] Riccardi C., Nicoletti I. (2006). Analysis of apoptosis by propidium iodide staining and flow cytometry. Nat. Protoc..

[22] Nguyen L.C., Yang D., Nicolaescu V., Best T.J., Gula H., Saxena D., Gabbard J.D., Chen S.N., Ohtsuki T., Friesen J.B. (2022). Cannabidiol inhibits SARS-CoV-2 replication through induction of the host ER stress and innate immune responses. Sci. Adv..

[23] Yoncheva K., Calleja P., Agueros M., Petrov P., Miladinova I., Tsvetanov C., Irache J.M. (2012). Stabilized micelles as delivery vehicles for paclitaxel. Int. J. Pharm..

[24] Taha E.I., Badran M.M., El-Anazi M.H., Bayomi M.A., El-Bagory I.M. (2014). Role of Pluronic F127 micelles in enhancing ocular delivery of ciprofloxacin. J. Mol. Liquids.

[25] Niu J., Yuan M., Chen C., Wang L., Tang Z., Fan Y., Liu X., Ma Y.J., Gan Y. (2020). Berberine-loaded thiolated Pluronic F127 polymeric micelles for improving skin permeation and retention. Int. J. Nanomed..

[26] Liu Z., Liu D., Wang L., Zhang J., Zhang N. (2011). Docetaxel-loaded Pluronic P123 polymeric micelles: In vitro and in vivo evaluation. Int. J. Mol. Sci..

[27] Fares A.R., ElMeshad A.N., Kassem M.A.A. (2018). Enhancement of dissolution and oral bioavailability of lacidipine via Pluronic P123/F127 mixed polymeric micelles: Formulation, optimization using central composite design and in vivo bioavailability study. Drug Deliv..

[28] Zaharieva M.M., Kroumov A.D., Dimitrova L., Tsvetkova I., Trochopoulos A., Konstantinov S.M., Berger M.R., Momchilova M., Yoncheva K., Najdenski H.M. (2019). Micellar curcumin improves the antibacterial activity of the alkylphosphocholines erufosine and miltefosine against pathogenic Staphyloccocus aureus strains. Biotechnol. Biotechnol. Equip..

[29] Chithrani B.D., Ghazani A.A., Chan W.C. (2006). Determining the size and shape dependence of gold nanoparticle uptake into mammalian cells. Nano Lett..

[30] Jiang W., Kim B.Y., Rutka J.T., Chan W.C. (2008). Nanoparticle-mediated cellular response is size-dependent. Nat. Nanotechnol..

[31] Canton I., Battaglia G. (2012). Endocytosis at the nanoscale. Chem. Soc. Rev..

[32] Rapoport N., Marin A., Luo Y., Prestwich G.D., Muniruzzaman M.D. (2002). Intracellular uptake and trafficking of Pluronic micelles in drug-sensitive and MDR cells: Effect on the intracellular drug localization. J. Pharm. Sci..

[33] Das R.P., Gandhi V.V., Singh B.G., Kunwar A. (2022). Balancing loading, cellular uptake, and toxicity of gelatin-pluronic nanocomposite for drug delivery: Influence of HLB of pluronic. J. Biomed. Mater. Res..

[34] Popovici C., Popa M., Sunel V., Atanase L.I., Ichim D.L. (2022). Drug delivery systems based on Pluronic micelles with antimicrobial activity. Polymers.

[35] Matarazzo A.P., Elisei L.M.S., Carvalho F.C., Bonfílio R., Ruela A.L.M., Galdino G., Pereira G.R. (2021). Mucoadhesive nanostructured lipid carriers as a cannabidiol nasal delivery system for the treatment of neuropathic pain. Eur. J. Pharm. Sci..

[36] Li K., Chang S.-L., Chang T.-R., You Y., Wang X.-D., Wang L.-W., Yuan X.-F., Tan M.-H., Wang P.-D., Xu P.-W. (2021). Inclusion complexes of cannabidiol with β-cyclodextrin and its derivative: Physicochemical properties, water solubility, and antioxidant activity. J. Mol. Liq..

[37] Čović A., Stipanelov Vrandečić N. (2021). Effect of poly(ethylene oxide) sample preparation on the results of thermogravimetric analysis. ST-OPEN.

[38] Mayr T., Grassl T., Korber N., Christoffel V., Bodensteiner M. (2017). Cannabidiol revisited. IUCrData.

[39] Pepic I., Lovric J., Hafner A., Filipovic-Grcic J. (2014). Powder form and stability of Pluronic mixed micelle dispersions for drug delivery applications. Drug Dev. Ind. Pharm..

[40] Shaarani S., Hamid S.S., Kaus N.H.M. (2017). The influence of Pluronic F68 and F127 nanocarrier on physicochemical properties, in vitro release, and antiproliferative activity of thymoquinone drug. Pharmacogn. Res..

